# Falcotentorial Meningiomas: Insights from Surgical Strategies and Clinical Outcomes

**DOI:** 10.3390/jcm13071963

**Published:** 2024-03-28

**Authors:** Arthur H. A. Sales, Christine Steiert, Simon P. Behringer, Marco Bissolo, Mazin Omer, Theresa Bettina Loidl, Jürgen Beck, Jürgen Grauvogel

**Affiliations:** Department of Neurosurgery, Medical Center, Faculty of Medicine, University of Freiburg, 79108 Freiburg, Germany; arthur.sales@uniklinik-freiburg.de (A.H.A.S.); christine.steiert@uniklinik-freiburg.de (C.S.); simon.behringer@uniklinik-freiburg.de (S.P.B.); marco.bissolo@uniklinik-freiburg.de (M.B.); mazin.omer@uniklinik-freiburg.de (M.O.); theresa.loidl@uniklinik-freiburg.de (T.B.L.); j.beck@uniklinik-freiburg.de (J.B.)

**Keywords:** falcotentorial meningioma, occipital interhemispheric approach, supracerebellar infratentorial approach, tentorial angle, skull base surgery, skull base meningioma

## Abstract

**Background**: Falcotentorial meningiomas are exceptionally uncommon tumors, presenting a challenge for neurosurgeons due to their close proximity to vital structures. Gross total resection represents the standard of treatment for these tumors. However, care must be taken when surgically approaching these lesions, since damaging neurovascular structures may cause unacceptable morbidity. Selecting the optimal surgical approach for each tumor is of paramount importance when treating these patients. **Methods**: The authors reviewed medical records to identify all patients with falcotentorial meningiomas who underwent resection at the University Hospital of Freiburg between January 2001 and December 2021. Clinical and imaging data, surgical management, and clinical outcomes were analyzed. **Results**: Falcotentorial meningiomas occurred in 0.7% (15 of 2124 patients) of patients with intracranial meningiomas. Of these 15 patients, 8 were female and 7 male. The occipital interhemispheric approach was used in nine patients, the supracerebellar infratentorial approach in five patients, and the retrosigmoidal approach in one patient. Three patients developed visual field deficits after surgical resection. Incomplete resection was significantly associated with tumor progression (*p* < 0.05). **Conclusions**: Individualized surgical strategies, guided by preoperative imaging and classification systems, play a crucial role in optimizing patient care. Among the available approaches, the occipital interhemispheric and supracerebellar infratentorial approaches are frequently employed and considered among the safest options for these tumors.

## 1. Introduction

Falcotentorial meningiomas are extremely rare tumors that represent a challenge for neurosurgeons because of the lesions’ depth and their proximity to important neurovascular structures [1,2,3].

Since the publication of the report on the successful removal of two pineal region meningiomas in 1937 by Araki et al., many authors have published small case series on patients with meningiomas affecting this region [1,2,3,4,5]. Here, it is important to emphasize that falcotentorial meningiomas and pineal region meningiomas are two distinct entities, since the latter must be originated from the arachnoid envelope of the pineal gland or from the velum interpositum without dural attachments [6,7]. Falcotentorial meningiomas originate by definition in the junction between the falx and tentorium and may grow in the quadrigeminal cistern, where the pineal gland is located.

The selection of the surgical approach to these tumors depends mainly on tumor location, especially regarding its position related to relevant vascular structures, as the straight sinus and the vein of Galen [4]. Asari et al. as well as Bassiouni et al. have proposed similar classification systems based on preoperative imaging that analyze the origin of the tumor and its growth pattern, which may help in the decision-making process regarding the best surgical approach [4,8].

Tumor location also influences clinical manifestations, since supratentorial tumors often compress the visual cortex causing visual deficits, while infratentorial tumors are often related to cerebellar symptoms and obstructive hydrocephalus.

Regarding clinical outcomes, previous studies have reported a rate of new postoperative deficits and complications between 10 and 50%, although a significant fraction of these deficits has been described as transient and resolved over the course of weeks after surgery [2,3,4,5,8,9,10].

The current work reports on our single-center experience in the last two decades, focusing on clinical manifestations, surgical aspects and clinical outcomes.

## 2. Material and Methods

### 2.1. Study Design, Patient Selection, and Data Collection

Two thousand one hundred twenty-four patients with intracranial meningiomas who were treated with surgical resection at the department of neurosurgery between January 2001 and December 2021 were screened for this retrospective cohort. Fifteen patients with falcotentorial meningiomas, as demonstrated by preoperative imaging and histopathological confirmation, were included in this study. Patients with meningiomas in other intracranial locations as well as patients with other tumor entities located in the falcotentorial junction have been excluded from this retrospective cohort.

Demographic, clinical, imaging, surgical, histopathological and outcome data were retrospectively collected from electronic medical records.

### 2.2. Volumetric Analysis, Vascular Features, and Tentorial Angle

Preoperative magnetic resonance imaging (MRI) was assessed by a physician who was blinded to clinical outcomes. Post-contrast T1-weighted images were utilized to determine the preoperative contrast-enhancing tumor volume and tumor diameter. Pre-contrast T1-weighted images were employed to evaluate the presence of hydrocephalus. Additionally, fluid-attenuated inversion recovery (FLAIR) sequences were used to estimate preoperative peritumoral edema. This was achieved by subtracting the contrast-enhancing tumor volume, as measured in T1-weighted sequences, from the hyperintense area measured by FLAIR sequences. Tumor progression was retrospectively determined based on a definitive increase in nodular enhancement observed on consecutive MRI scans or an increase in tumor volume of 15% or greater as described by Przybylowski et al. [11].

The assessment of contrast-enhanced tumor volume, as well as FLAIR hyperintense areas, was performed using a semi-automated volumetric measurement technique (SmartBrush, Brainlab AG, Feldkirchen, Germany).

Magnetic resonance venography (MRV) and computer tomography venography (CTV) were employed to assess the displacement and patency of relevant vascular structures such as the straight sinus and the vein of Galen in some cases.

The tentorial angle was measured at the line joining the nasion to the tuberculum sellae, crossing the tentorium in the midsagittal plane as described by Kao et al. and Zhao et al. [9,12]. Both volumetric measures and the tentorial angle were measured three times, and the mean value was analyzed in order to improve accuracy.

### 2.3. Statistical Analysis

Data are presented as mean (standard deviation), number of patients (percentage), or median (interquartile range). We used the independent *t*-test to compare numerical data and Pearson’s Chi-square test to compare categorical variables. Predictive factors for clinical outcomes were investigated by means of logistic regression. Descriptive data analysis, independent *t*-test, Pearson’s Chi-square test, and logistic regression were performed using IBM SPSS Statistics version 25.0 (SPSS Inc., IBM Corp., Armonk, NY, USA).

## 3. Results

### 3.1. Clinical Characteristics

Two thousand one hundred twenty-four patients with intracranial meningiomas were screened for this retrospective cohort, of whom 15 were included and analyzed. Of these 15 patients, 8 were female and 7 male. Mean age was 55.6 (±11.3). See Table 1.

Status (KPS) was 90 (IR: 80–100). Twelve patients presented clinical symptoms at the time of diagnosis, while three patients were asymptomatic. Six patients presented headaches, two seizures, three motor deficits, six gait disturbances, one sensory deficits, and three cerebellar ataxia and dysmetria (Table 2). Of the three patients with preoperative motor deficits, two presented moderate hemiparesis according to the Medical Research Council scale (MRC) 3/5, while one patient had a mild hemiparesis MRC 4/5. The mean interval between first clinical manifestation and surgical resection was 92.3 days (±50.5).

Twelve patients had received dexamethasone prior to surgical treatment, and one patient was treated with anticonvulsants due to preoperative seizures. Only one patient had received radiosurgery before surgical resection.

### 3.2. Preoperative Imaging Data

**Figure 1:**
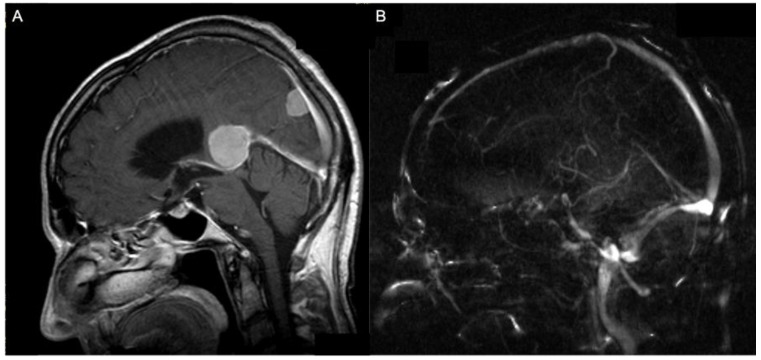
A 74-year-old patient with an anterior (Asari), type I (Bassiouni) falcotentorial meningioma (**A**) and the corresponding MR-angiographic examination showing a normal patency of the vein of Galen (**B**).

Thirteen patients had preoperative MRI scans, while two patients had only preoperative CT scans. Six patients had preoperative MR angiography in order to demonstrate displacement or encasement of major vessels by the tumor mass, while one patient had preoperative CT angiography and another patient a digital subtraction angiography with the same purpose. Five patients presented displacement of the straight sinus and four of the vein of Galen. In one patient, the straight sinus was infiltrated by the tumor mass; in two, the superior sagittal sinus was invaded by the tumor (Figure 1).

**Figure 2:**
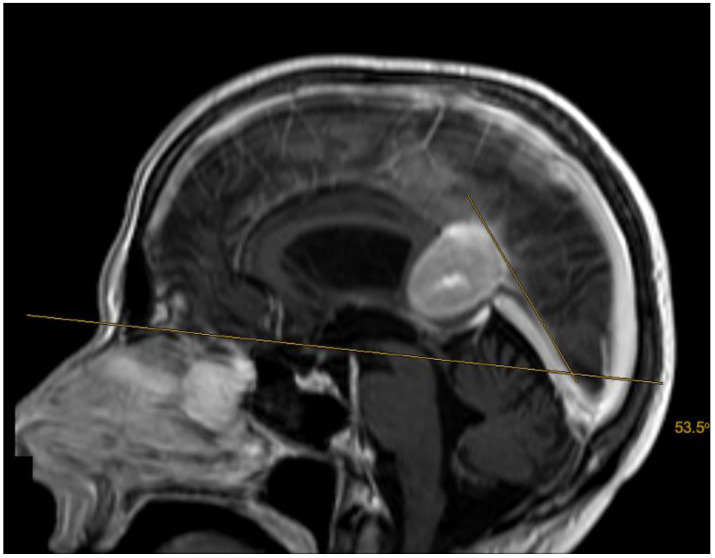
A 51-year-old patient with an anterior (Asari), type I (Bassiouni) falcotentorial meningioma. The tentorial angle was measured as described by Kao et al. and Zhao et al. [9,12].

No patient underwent preoperative tumor embolization. Four patients exhibited non-communicating hydrocephalus in the preoperative imaging. The mean interval between the first preoperative imaging and surgical resection was 35.3 days (±30.9). The mean preoperative tumor volume was 17.8 cm^3^ (±17.2), the mean preoperative tumor edema was 21.9 cm^3^ (±38.0). The mean tentorial angle was 49.6° (±6.2) (Figure 2).

**Figure 3:**
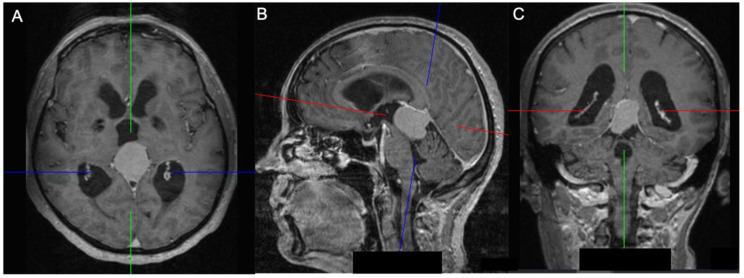
A 55-year-old patient with an inferior (Asari), type II (Bassiouni) falcotentorial meningioma: axial (**A**), sagittal (**B**) and coronal view (**C**) showing an obstructive hydrocephalus caused by the mass effect in the posterior cranial fossa. The blue line represents the anteroposterior axis, while the red line represents the superoinferior axis, and the green line represents the laterolateral axis.

**Figure 4:**
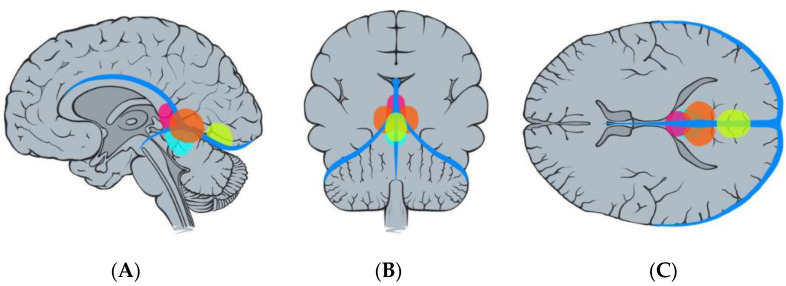
Illustration demonstrating the classification of falcotentorial meningiomas according to Bassiouni et al. [4], type I (pink), type II (blue), type III (orange), and type IV (yellow): sagittal (**A**), coronal (**B**), and axial (**C**) view.

Regarding tumor origin and its direction of growth according to Bassiouni et al., seven tumors were classified as type I (tumor origin between the leaves of the falx immediately above the junction of the great vein of Galen with the straight sinus), five as type II (tumor origin underneath the tentorium near the junction of the vein of Galen with the straight sinus), one as type III (tumor origin paramedian the tentorial incisura, lateral to the vein of Galen and straight sinus), and two as type IV (tumor origin in the falcotentorial junction along the straight sinus) [4]. See Figure 3 and Figure 4.

**Figure 5:**
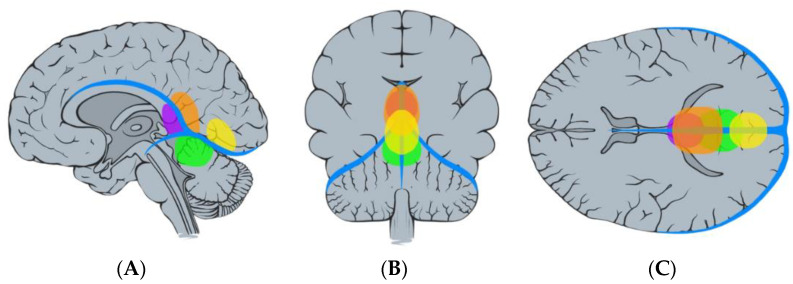
Illustration demonstrating the classification of falcotentorial meningiomas according to Asari et al. [8]. Anterior (purple), superior (orange), posterior (yellow), and inferior (green): sagittal (**A**), coronal (**B**), and axial (**C**) view.

In regard to direction of tumor extension as described by Asari et al., seven tumors were classified as anterior type (growth towards the anterior extension between the inferior sagittal sinus and the great vein of Galen), five as inferior type (growth towards the inferior extension between the great vein of Galen and the straight sinus), and three as posterior type (growth towards the posterior extension along the straight sinus) [8]. See Figure 5.

### 3.3. Surgical Strategy and Intraoperative Data

In this series, three approaches were used to access the falcotentorial meningiomas: occipital interhemispheric approach in nine patients, supracerebellar infratentorial approach in five patients, and retrosigmoidal approach in one patient (Table 2).

Six patients with a type I falcotentorial meningioma, one with a type III, and two with a type IV tumor were operated by means of an occipital interhemispheric approach. One patient with a type I tumor and four with a type II tumor were treated by means of a supracerebellar infratentorial approach. The only tumor resected by the retrosigmoidal approach was a type II tumor.

Regarding the classification by Asari et al., six patients with an anterior tumor and three patients with a posterior tumor were treated by the occipital interhemispheric approach, while four patients with an inferior tumor and one with an anterior tumor were treated by the supracerebellar infratentorial approach. The only patient operated by means of a retrosigmoid approach had an inferior tumor.

Gross total resection (Simpson grades I and II) was achieved in 13 patients, while 1 patient had Simpson grade III and another a Simpson grade IV resection (Table 3).

Seven patients received intraoperative mannitol in order to improve brain relaxation. Two patients had an intraoperative injury of the inferior sagittal sinus, while only one patient had an intraoperative injury of the transverse sinus. No patient presented intraoperative injuries of the vein of Galen or straight sinus.

There was no relation between tentorial angle and resection grade in this series. The mean duration of surgery was 273 min (±100.0), while the mean intraoperative blood loss was 688 mL (±447.2).

### 3.4. Histological Features

Ten patients had meningothelial meningiomas (CNS WHO grade 1), two patients had transitional meningiomas (CNS WHO grade 1), one patient had a fibrous meningioma (CNS WHO grade 1), and two patients had atypical meningiomas (CNS WHO grade 2). No CNS WHO grade 3 tumor was reported.

### 3.5. Clinical Outcomes

The mean patient follow-up was 71.2 months (±60.4). The median postoperative KPS was 90 (IR 80–90). Three patients developed new neurological deficits: two patients developed homonymous hemianopsia, and another quadrantanopsia. These deficits were still present in all three patients three months after surgical resection.

Regarding postoperative complications, one patient presented a superficial wound healing disorder and was treated with antibiotics only, while another had a postoperative cerebrospinal fluid leak associated with meningitis. Two patients received postoperative radiotherapy: one patient had an incomplete resection (Simpson grade III), while another had an atypical meningioma CNS WHO grade 2.

Three patients presented tumor progression. Of these three patients, two had progression after one year of tumor resection, while one patient recurred seven months after surgery. In addition, two of these patients had a CNS WHO grade 1 tumor, while one patient had a CNS WHO grade 2 tumor. Incomplete resection was significantly associated with progression rate (Pearson’s Chi-square test; *p* < 0.05).

## 4. Discussion

In this study, we have reported our experience in treating 15 patients with falcotentorial meningiomas. Patients with falcotentorial meningiomas represented 0.7% of all patients with intracranial meningiomas treated at our institution between January 2001 and December 2021, which is consistent with the previously reported incidence of 0.3–1.1% [7,9,10,13].

Regarding clinical manifestations, headache and gait disturbances were the most common symptoms (40% of incidence each). This fact was also confirmed by previous studies [2,8,10]. There was no significant association between headache and obstructive hydrocephalus or tumor location. The incidence of gait disturbance and obstructive hydrocephalus was significantly higher in tumors located predominantly infratentorially, as expected (Pearson’s Chi-square test; *p* < 0.05). These tumors are mostly classified as inferior type by Asari and Type II by Bassiouni. Upward gaze palsy due to damage of the vertical gaze center in the rostral part of the midbrain was not observed in this series. Homonymous hemianopsia was identified only in two patients harboring posterior meningiomas. The mean age (55.6 years) in our series was similar to that in previous studies (51.3 years–59.8 years), while the significantly higher incidence of disease in female patients reported in previous series was not reproduced in this study (53% vs. 47%) [7,9,13,14,15].

Concerning radiological features, MR angiography was the main diagnostic tool used to investigate patency and displacement of relevant vascular structures in our study as well as in previous series [2,4]. In this series, an occlusion of the vein of Galen was not observed, while the straight sinus was invaded by the tumor mass in one patient. Bassiouni et al. have described that the straight sinus was occluded or stenosed in 2 of 13 patients in their series, while the deep venous system was patent in both patients with a type IV tumor [4]. Asari et al. reported that the straight sinus and the posterior half of the vein of Galen was occluded or severely stenosed in three of seven patients in their retrospective study. In addition, they could not show a relevant association between tumor location and vascular occlusion since each patient harbored a different tumor type (inferior, anterior, and posterior) [8]. Liu et al. reported a surprisingly high straight sinus occlusion rate of 79.6% (39 of 49 patients) according to preoperative MR venography studies [5]. CT venography was performed only in one patient in our series, whereas another patient underwent preoperative digital subtraction angiography. It is important to emphasize that the latter method enables preoperative embolization of tumors when indicated. However, in this series, no patient underwent preoperative embolization.

According to the Asari classification, 7 of 15 tumors were classified as anterior type, 5 as inferior type, and 3 as posterior type. Superior tumors were not observed in this series. This was also reported by Asari et al. in their classic study that established this classification system [8]. In the study by Zhao et al., superior tumors represented 25% of patients (4/16) [9]. According to the radiological classification of Bassiouni 7 of 15 tumors were classified as type I, 5 as type II, 1 as type III, and 2 as type IV.

Zhao et al. stated that patients with a steep tentorium tended to have unfavorable resection rates following a supracerebellar infratentorial approach [9]. Liu et al. related that a steeper tentorial angle was associated with occipital lobe damage risk in patients undergoing tumor resection with the occipital transtentorial approach [16]. These results could not be reproduced in our retrospective cohort. We did not find a significant association between tentorial angle and resection rate or risk of occipital lobe damage. Therefore, we do not recommend an approach selection based on tentorial angle only. Given that a steep tentorial angle has a negative impact on outcomes for both the supracerebellar infratentorial and occipital transtentorial approaches, it should not be used as a determining factor for choosing one approach over the other.

In terms of surgical approach selection, the supracerebellar infratentorial (5 of 15 patients) and occipital interhemispheric (9 of 15 patients) approaches were mainly used. This fact was echoed by many authors in their retrospective series [2,4,5,7,8,9,10]. Only one patient with an inferior tumor was operated with a retrosigmoid approach.

The occipital interhemispheric approach, as we know it today, was initially described by Poppen in the 1960s and later refined by Yasargil in the 1980s [17,18,19,20,21]. In our series, we employed the occipital interhemispheric approach for the resection of predominantly supratentorial tumors, both posterior and anterior types. This approach was not used for only one anterior tumor, primarily due to a significant portion of the tumor being located beneath the tentorium. In addition, all type I, type IV, and type III tumors were resected using this surgical route. This approach offers several advantages, such as the minimal presence of bridging veins between the occipital lobe and the superior sagittal sinus, as well as the possibility to release cerebrospinal fluid (CSF) by cannulating the posterior ventricular horn, allowing for a more gentle retraction of the occipital lobe [4]. Another positive aspect of this approach is the gravity-assisted retraction of the ipsilateral occipital lobe [9]. We have not employed the occipital interhemispheric approach for the resection of inferior tumors due to the potential for direct interference with the vein of Galen and its tributaries, which could impede surgical access. However, in theory, inferior tumors could also be safely resected using this approach if the preoperative MR venography reveals an occluded straight sinus, since the approach could be supplemented by a transtentorial maneuver. Ding et al. reported favorable outcomes by combining endoscopic and microscopic surgery in patients with pineal region meningiomas. They stated that the utilization of a combined technique effectively eliminates microscopic blind spots, addressing the limitations of the traditional occipital transtentorial approach [22]. The most common complication associated with the occipital interhemispheric approach is a visual field deficit, as observed in two of nine patients operated with this approach in our series. Lopez-Gonzalez et al. reported no visual complications with a retractorless interhemispheric approach in their case series, which included patients with falcotentorial meningiomas and cerebellar tumors [23].

The supracerebellar infratentorial approach was described by Krause and Oppenheim in 1913 and was posteriorly refined by Stein [24,25,26,27,28]. This approach was used mainly for the resection of tumors located predominantly infratentorially in this series. Four of five inferior and type II tumors were resected with this technique. This approach is particularly advantageous for treating velum interpositum meningiomas originating from the ventral half of the tela choroidea, as they displace the internal cerebral veins dorsally [7]. Only one inferior tumor was resected with a retrosigmoid approach in this series. Coincidently, a gross total resection (Simpson resection grade I or II) was not achieved in this case. One anterior tumor was resected with the supracerebellar infratentorial approach, and a gross total resection was not achieved due to the adhesions to the deep venous system. A second-stage surgery via occipital interhemispheric approach may be performed in selected cases, as described by Zhao et al. [9]. Staged surgery was not performed in our patients, and residual tumors were treated by means of radiotherapy. The combination of both approaches was reported for the resection of a large falcotentorial meningioma with minimal brain retraction, preservation of the vein of Galen and straight sinus, and no postoperative neurological deficits [29]. The most common complication associated with the supracerebellar infratentorial approach is the risk of air embolism in patients operated in a semi-seated position. Therefore, some surgeons perform this approach with the patient in the park bench position [5]. In our series, five patients were operated in the semi-seated position, nine patients in the prone position, and one patient in the supine position.

Other surgical approaches used to treat falcotentorial meningiomas are: the anterior interhemispheric approach, torcular approach, parietal transventricular approach, bioccipital interhemispheric approach, parietal transcallosal approach, and transtemporal transventricular approach [4,9]. In addition, gamma knife radiosurgery represents a valuable therapeutic approach for patients diagnosed with small falcotentorial meningiomas or those with progressive residual tumors following surgical resection [30].

Regarding clinical outcomes, three patients developed new neurological deficits. Three patients presented new visual field deficits that persisted three months after surgery. Visual complications are the most common complication in patients with falcotentorial meningiomas, according to previous studies [4,5,8]. There was no surgical fatality in this study. We have not found a significant association between tumor location or surgical approach and new neurological deficits. Goto et al. reported differences in surgical complexity between superior and inferior tumors. They stated that the higher complexity of resection of inferior tumors may be linked to the interaction of the arachnoid membrane with deep veins, the brainstem, and the tumor itself. In superior tumors, the arachnoid membrane between the tumor and the quadrigeminal cistern protects deep veins, facilitating tumor dissection, while inferior tumors often directly compress and adhere to critical vascular structures in the quadrigeminal cistern [2].

Postoperative radiotherapy was performed in patients with CNS WHO grade 2 tumors as well as in patients with incomplete resection of tumor mass. Incomplete tumor resection was significantly associated with progression rate (*p* < 0.05). However, given the small sample size of this study, care must be taken when interpreting this particular result. Larger prospective studies are necessary in order to evaluate this outcome.

## 5. Conclusions

In conclusion, falcotentorial meningiomas represent a rare and challenging subset of intracranial tumors. Our retrospective analysis provides valuable insights into their clinical presentations, surgical management, and clinical outcomes. Tailored surgical approaches guided by preoperative imaging and classification systems are critical for optimizing patient care. While achieving a gross total resection is considered the standard of care for these patients, it is of paramount importance to prioritize the preservation of critical vascular structures, such as the vein of Galen and straight sinus, as well as to minimize the risk of new neurological deficits when managing these patients. Further research and collaborative efforts within the medical community will continue to advance our understanding of these unique tumors and improve patient outcomes.

## Figures and Tables

**Table 1:** Clinical and surgical data of patients with falcotentorial meningiomas.

Case	Age	Sex	Asari Type	Bassiouni Type	Surgical Approach	Simpson Resection Grade	Pathology	CNS WHO Grade	Progression
1	51	M	Posterior	III	Occipital interhemispheric	2	Atypical meningioma	2	No
2	55	F	Posterior	IV	Occipital interhemispheric	2	Meningothelial meningioma	1	No
3	51	F	Anterior	I	Occipital interhemispheric	2	Meningothelial meningioma	1	No
4	51	M	Inferior	II	Supracerebellar infratentorial	1	Transitional meningioma	1	No
5	75	M	Inferior	II	Retrosigmoid	3	Meningothelial meningioma	1	Yes
6	71	M	Posterior	IV	Occipital interhemispheric	2	Meningothelial meningioma	1	Yes
7	55	F	Inferior	II	Supracerebellar infratentorial	2	Meningothelial meningioma	1	No
8	69	F	Anterior	I	Supracerebellar infratentorial	2	Fibrous meningioma	1	No
9	43	M	Anterior	I	Occipital interhemispheric	2	Meningothelial meningioma	1	No
10	45	F	Inferior	II	Supracerebellar infratentorial	2	Meningothelial meningioma	1	No
11	46	F	Anterior	I	Occipital interhemispheric	2	Transitional meningioma	1	No
12	46	F	Inferior	II	Supracerebellar infratentorial	2	Meningothelial meningioma	1	No
13	74	M	Anterior	I	Occipital interhemispheric	2	Meningothelial meningioma	1	No
14	59	M	Anterior	I	Occipital interhemispheric	1	Meningothelial meningioma	1	No
15	44	F	Anterior	I	Occipital interhemispheric	4	Atypical meningioma	2	Yes

**Table 2:** Patient characteristics.

General data	Age (years)	55.6 (±11.3)
	Sex (male/female)	7/8
Preoperative clinical data	Preoperative Karnofsky (%)	90 (IR: 80–100)
	Headache	6
	Seizures	2
	Motor deficits	3
	Gait disturbances	6
	Sensory deficits	1
	Visual field deficits	3
	Cerebellar ataxia	3
Radiological data	Preoperative tumor volume (cm^3^)	17.8 (±17.2)
	Preoperative tumor edema (cm^3^)	21.9 (±38.0)
	Infiltration of the straight sinus	1
	Infiltration of the superior sagittal sinus	2
	Displacement of the straight sinus	5
	Displacement of the vein of Galen	4
	Hydrocephalus	4
	Tentorial angle	49.6 (±6.2)
Surgical data	Occipital interhemispheric approach	9
	Supracerebellar infratentorial approach	4
	Retrosigmoid approach	1
	Gross total resection (Simpson grades I and II)	13
	Subtotal resection (Simpson III and IV)	2
	Mean duration of surgery (min)	273 (±100)
	Mean intraoperative blood loss (mL)	688 (±447.2)
Histological features	CNS WHO grade 1	13
	CNS WHO grade 2	2
	Meningothelial meningioma	10
	Transitional meningioma	2
	Fibrous meningioma	1
	Atypical meningioma	2
Clinical outcomes	Postoperative Karnofsky (%)	90 (IR:80–90)
	Postoperative neurological deficits	3
	Visual field deficits	3
	Postoperative complications	2
	Postoperative radiotherapy	2
	Tumor progression	3
	Mean follow-up (months)	71.2 (±60.4)

**Table 3:** Surgical approach and tumor classification after Asari and Bassiouni.

	Type I	Type II	Type III	Type IV	Anterior	Inferior	Posterior
Occipital interhemispheric	6	0	1	2	6	0	3
Supracerebellar infratentorial	1	4	0	0	1	4	0
Retrosigmoid	0	1	0	0	0	1	0

## Data Availability

The raw data supporting the conclusions of this article will be made available by the authors on request.
